# Brand Awareness via Online Media: An Evidence Using Instagram Medium with Statistical Analysis

**DOI:** 10.1155/2022/2739685

**Published:** 2022-01-10

**Authors:** Mi Yantian, Zubair Ahmad, Ibrahim Alkhairy, Hassan Alsuhabi, Morad Alizadeh, M. R. Mouhamed

**Affiliations:** ^1^School of Communication, Harbin Normal University, Harbin City, Heilongjiang Province, China; ^2^Department of Statistics, Yazd University, P.O. Box 89175-741, Yazd, Iran; ^3^Department of Mathematics, Al-Qunfudah University College, Umm Al-Qura University, Mecca, Saudi Arabia; ^4^Department of Statistics, Faculty of Intelligent Systems Engineering and Data Science, Persian Gulf University, Bushehr 75169, Iran; ^5^Faculty of Science, Mathematics Department, Helwan University, Cairo, Egypt

## Abstract

Online marketing refers to the practices of promoting a company's brand to its potential customers. It helps the companies to find new venues and trade worldwide. Numerous online media such as Facebook, YouTube, Twitter, and Instagram are available for marketing to promote and sell a company's product. However, in this study, we use Instagram as a marketing medium to see its impact on sales. To carry out the computational process, the approach of linear regression modeling is adopted. Certain statistical tests are implemented to check the significance of Instagram as a marketing tool. Furthermore, a new statistical model, namely a new generalized inverse Weibull distribution, is introduced. This model is obtained using the inverse Weibull model with the new generalized family approach. Certain mathematical properties of the new generalized inverse Weibull model such as moments, order statistics, and incomplete moments are derived. A complete mathematical treatment of the heavy-tailed characteristics of the new generalized inverse Weibull distribution is also provided. Different estimation methods are discussed to obtain the estimators of the new model. Finally, the applicability of the new generalized inverse Weibull model is established via analyzing Instagram advertising data. The comparison of the new distribution is made with two other models. Based on seven analytical tools, it is observed that the new distribution is a better model to deal with data in the business, finance, and management sectors.

## 1. Introduction

Advertising means a way of business communication between the company/business firm and its present and prospective audience. It provides information about the business firms, their brand qualities, price, place of availability, etc. For a better business deal, advertisement is essential for both the company and the customers. However, it is more fruitful for the company to reach maximum customers [1].

Advertising is an effective and useful step of marketing to promote a specific brand. Marketing is a collection of the process that involves designing the brand, creation, research, and investigation about how to promote the products/services to the potential and target and customers [2]. An effective marketing program, also known as a marketing plan or marketing strategy, helps to define the price and quality of the product. Several methods have been suggested for marketing a company's brand. However, marketing through online media is very fruitful to reach the maximum audience [3].

Numerous online platforms such as YouTube, Instagram, Facebook, Pinterest, Twitter, and Flickr are available for online marketing; see Dwivedi et al. (2015). Among the available online platforms for marketing, Instagram is one of the most beneficial tools for online marketing [4].

A decade ago, Instagram was founded by Michel Kriger (a software engineer) and Kevin Systrom (a former Google employee). In April 2012, Facebook bought Instagram for $1 billion. It is one of the most influential and biggest social media platforms (SMPs). In June 2018, this platform had hit one thousand million monthly active users [5]. Due to a large number of active users, it is a very useful platform for online marketing; see Salleh et al. [6] and Yu et al. [7].

In this work, we test the significance of online media on the sales of certain products. For this activity, we choose the Instagram medium among the well-known online platforms. We use a simple linear regression (SLR) model to check the significance of the Instagram medium. Two well-known statistical tests such as the (i) *t*-test and (ii) *F*-test, along with the correlation test (CT) are considered to perform the regression analysis (RA).

In addition to the RA, a new flexible statistical distribution (SD) is introduced to model the Instagram sales data. The proposed SD may be called a new generalized inverse Weibull (NIG-Weibull) model. The NGI-Weibull is very flexible and offers a close fit to Instagram sales data.

## 2. Regression Analysis

Within this section, we provide the RA to see the impact and usefulness of advertising on sales using the Instagram medium. Furthermore, we apply the *t*-test statistic and *F*-test statistic to test a hypothesis about the significant role of Instagram advertising in the business sector.

### 2.1. Simple Linear Regression Model

The SLR model to describe the relationship between Instagram advertising and sales has the following form:

(1) $$\begin{array}{c} Y=\lambda_{0}+\lambda_{1}\text{ Instagram }+\varepsilon . \end{array}$$

By implementing the RA technique, we observe that *λ*_0_=5.1030, interpreted as the expected dollar sales (in 1000 s). So, for allocating no advertising budget on Instagram, the expected sale is 5.1030*∗*1000=5103. The slope of the regression model (regression coefficient) presented in equation (1) is 0.193 5, indicating an increment of 193 (0.193 5 *∗* 1000) units in the sales. This fact shows that by spending money on Instagram medium, the expected sale is 5.103 0 + 0.193 5 *∗* 1000 = 198.603, representing a sale of $198 603. The estimated regression model concerning equation (1) is given by

(2) $$\begin{array}{c} Y=5.1030+0.1935\text{ Instagram}. \end{array}$$

**Figure 1:**
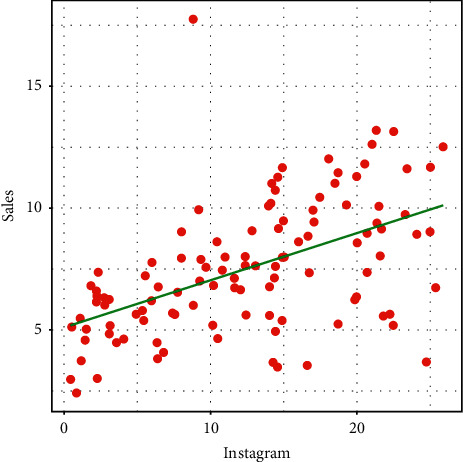
Relationship between Instagram advertising and sales.

A visual display (graphical illustration) of the positive linear relationship between Instagram medium and sales is presented in Figure 1. From the visual display in Figure 1, we observe that spending money on Instagram advertising is very fruitful and helps to increase the sale.

### 2.2. Hypothesis Testing

Here, we implement a statistical technique called hypothesis testing to check the significance and usefulness of Instagram advertising on sales. To perform the hypothesis testing, we adopt the *t*-test and *F*-test. The null hypothesis (NH) usually represented by (*H*_0_) and alternative hypothesis (AH) usually represented by (*H*_*A*_) can be formulated as (HTML translation failed)  = *Instagram advertisement has no impact on sales* VS *H*_*A*_  = *Instagram advertisement has an impact on sales*.

#### 2.2.1. *t*-Test

To carry out the numerical computation using the *t*-test, we need to find whether the estimate of the regression coefficient (RC) *λ*_1_ is far from 0 or not. If the standard error (SE) of the estimate of the RC *λ*_1_ is very small, then we have sufficient evidence to reject NH *H*_0_. After implementing the *t*-test, a summary of the numerical analysis is reported in Table 1.

From the results in Table 1, we can see that for (i) *λ*_0_, the value of *t*-statistic is 11.389 and *p* value is less than 2e-16, and for (ii) *λ*_1_, the value of *t*-statistic is 6.259 and *p* value is less than 8.01*e* − 09. As we see that the value of the *t*-statistic (for *λ*_0_ and *λ*_1_) is far from zero and the *p* value is less than 0.05, therefore, we have enough evidence against *H*_0_, and so, we reject it.

#### 2.2.2. *F*-Test

In this part, we implement the *F*-test to see the impact/significance of Instagram advertising (*X*) on sales (*Y*). A larger value of *F*-test statistic indicates a significant impact of *X* on *Y*. After carrying out the computation process, the numerical results are summarized in Table 2. From the presented results in Table 2, we have *F*-test = 39.17 with *p* value = 8.011*e* − 09. From the results in Table 2, we conclude that spending an advertising budget on the Instagram medium will increase the sale.

The *R* square (*R*^2^) is an important statistical tool for measuring the fit of the underlined regression model. It deals with the linear relationship between the response variable (sale in this study) and the explanatory variable (Instagram medium in this study). The value of (*R*^2^) ranges between 0 and 1. Its value near to 1 indicates the best fit, and a value near to 0 indicates the poor fit. Corresponding to Instagram advertising data, we observe *R*^2^=0.2594. So, using Instagram as an advertising tool, the sale can be increased up to 25.94%.

### 2.3. Correlation Test

**Figure 2:**
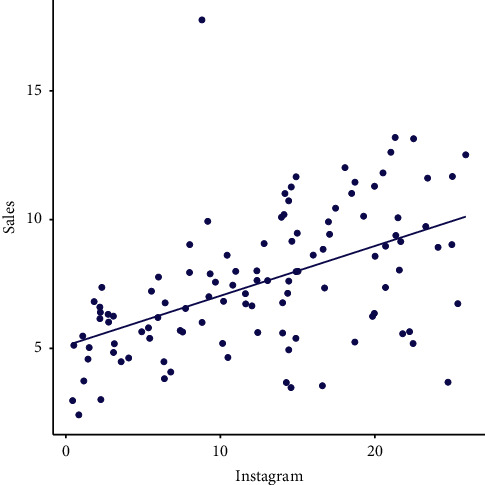
Visual sketching of the numerical results related to CT.

The CT is an important statistical approach to evaluate the association between two variables. In this study, we have considered two variables (Instagram advertising and sales). To check the correlation between *X* and *Y*, we consider the Pearson correlation test to check the linear dependency between *X* and *Y*. The Pearson correlation coefficient (PCC) expressed by *r* is calculated as follows:

(3) $$\begin{array}{c} r=\frac{\sum_{i=1}^{p}\left(\text{Instagarm }-M_{\text{Instagarm}}\right)\left(\text{Sales }-M_{\text{Sales}}\right)}{\sum_{i=1}^{p}{\left(\text{Instagarm }-M_{\text{Instagarm}}\right)}^{2}{\left(\text{Sales }-M_{\text{Sales}}\right)}^{2}}, \end{array}$$

where *M*_Instagram_ and *M*_Sales_ represent the mean values of Instagram advertising and sales data, respectively. If the *p* value is <0.05, then it shows a significant correlation between Instagram medium and sales. After applying the CT, we have *r*=0.5471673, showing a positive relationship (PR) between Instagram advertising and sales; see Figure 2. Corresponding to this test, we observe that the value of Spearman's rank correlation is 100 445 and the *p* value is 6.208*e* − 10. As the *p* value is <0.05, therefore, we reject *H*_0_, which states that Instagram advertisement has no significant impact on sales.

## 3. Statistical Modeling

After performing the RA, we move forward and introduce a novel SD for dealing with the Instagram advertising data. This section is organized into six different subsections: (a) the very first part of the section deals with the introduction of the methodology used to obtain the new model, namely NGI-Weibull (new generalized inverse Weibull) model, (b) the new model is fully described in the second subsection, (c) the heavy-tailed (HT) characteristics of the NGI-Weibull are provided in the third subsection, (d) some mathematical properties are obtained in the fourth subsection, (e) the estimators of the NGI-Weibull are obtained in the fifth subsection, and finally (d) the sixth part of this section is devoted to analyzing the Instagram advertising data.

### 3.1. Literature and Statistical Methodology

During the last couple of decades, serious developments have been made in distribution theory (DT) to propose new flexible statistical distributions or families of distributions. The statistical distributions play a useful role to model data in numerous areas such as (a) health sector, (b) finance sector, and (c) reliability engineering. Due to the applicability of the statistical distributions in applied sectors, numerous extensions and modifications of the existing distributions have been proposed; see Tahir and Cordeiro [8].

In the health sector, Wahed et al. [9] proposed a new generalized (NG) form of the Weibull distribution (WD) for modeling breast cancer data. Zhu et al. [10] used the WD for modeling the survival times of patients with gastric cancer. Jan et al. [11] applied the transmuted exponentiated IW (TEIW) distribution of the survival times of patients having bladder cancer. Yoosefi et al. [12] used the exponentiated Weibull (EW) distribution for modeling the survival times of colorectal cancer patients. Mohammed et al. [13] studied a new modified (NM) form of the WD and analyzed the bladder cancer data.

In the finance sector, Nadarajah and Kotz [14] applied the modified Weibull (MW) for asset returns. Bakar et al. [15] used the composite models for modeling loss data. Bhati and Ravi [16] applied the generalized log-Moyal (GLM) model to the Norwegian fire insurance loss data. Punzo and Bagnato [17] applied the Laplace scale mixtures (LSMs) to data related to cryptocurrencies.

In reliability engineering, Sarhan and Zaindin [18] used the modified Weibull (MW) distribution to model the lifetime of electronic devices. Almalki and Yuan [19] introduced a new modified Weibull (NMW) model for dealing with reliability data. Singh [20] proposed the additive Perks–Weibull (APW) distribution form modeling the Arset data. Okasha et al. [21] analyzed the failure time data by introducing the extended inverse Weibull (EIW) distribution.

As per the studies of Cooray and Ananda [22], Gebizlioglu et al. [23], Scollnik and Sun [24], Bernardi et al. [25], Adcock et al. [26], Miljkovic and Grun [27], Bhati and Ravi [16], and Punzo [28], the HT distributions are very competent for dealing with the data in business, finance, management, and other connected sectors.

According to the findings of Beirlant et al. [29], a statistical model is said to possess the HT characteristics, if its survival function (SF) $\bar{T}\left(z;\Phi \right)=1-T\left(z;\Phi \right)$ satisfies

(4) $$\begin{array}{c} \underset{z⟶\infty }{\text{lim}}=\exp\left\{mz\right\}\bar{T}\left(z;\Phi \right)=\infty , \end{array}$$

where *m* > 0.

An important property of the HT models is the regular variational property (ReVaPr); see Resnick [30]. A model is termed as a regular varying (ReVa) model, if it obeys

(5) $$\begin{array}{c} \underset{z⟶\infty }{\text{lim}}\;\frac{1-T\left(mz;\Phi \right)}{1-T\left(z;\Phi \right)}=m^{-a}, \end{array}$$

where *a* ∈ {0, *∞*}.

In this work, we move a step further and contribute a new HT model to the literature of DT. Consider that the distribution function (DF) *T*(*z*; Φ) of the inverse Weibull (IW) model is given by

(6) $$\begin{array}{c} T\left(z;\Phi \right)=e^{-\lambda_{2}/z^{\lambda_{1}}},\;z,\lambda_{1},\lambda_{2}>0, \end{array}$$

with probability density function (PDF) *t*(*z*; Φ) given by

(7) $$\begin{array}{c} t\left(z;\Phi \right)=\frac{\lambda_{1}\lambda_{2}}{z^{\lambda_{1}+1}}e^{-\lambda_{2}/z^{\lambda_{1}}},\;z,\lambda_{1},\lambda_{2}>0. \end{array}$$

Recently, Wang et al. (2021) proposed a new generalized family of distributions via the DF *K*(*z*; *λ*, *ϕ*), which is given by

(8) $$\begin{array}{c} K\left(z;\lambda ,\Phi \right)=1-\frac{{\left(\bar{T}\left(z;\Phi \right)\right)}^{\lambda }}{e^{T\left(z;\Phi \right)}},\;z\in \mathbb{R},\lambda >0,\Phi \in \mathbb{R}, \end{array}$$

where $\bar{T}\left(z;\Phi \right)=1-T\left(z;\Phi \right)$. The PDF *k*(*z*; *λ*, *ϕ*) associated with equation (8) is as follows:

(9) $$\begin{array}{c} k\left(z;\lambda ,\Phi \right)=t\left(z;\Phi \right){\left[1-T\left(z;\Phi \right)\right]}^{\lambda -1}\frac{\left[\left(1+\lambda \right)-T\left(z;\Phi \right)\right]}{e^{T\left(z;\Phi \right)}},\;z\in \mathbb{R}, \end{array}$$

where *t*(*z*; Φ)=(d/d*z*)*T*(*z*; Φ).

We combine the DF of the IW model provided in equation (6) with the DF expressed in equation (8) to generate and study a new model, namely a NGI-Weibull model. The HT characteristics and behaviors of the NGI-Weibull model are obtained. The parameters of the NG-Weibull are estimated via different estimation approaches. Finally, after carrying out the mathematical work, real-life data are analyzed.

### 3.2. A NGI-Weibull Distribution

Suppose *Z* follows the three-parameter NGI-Weibull model with two shape parameters (*λ*, *λ*_1_ > 0) and one scale parameter (*λ*_2_ > 0), then its DF *K*(*z*; *λ*, Φ) is given by

(10) $$\begin{array}{c} K\left(z;\lambda ,\Phi \right)=1-\frac{{\left(1-e^{-\lambda_{2}/z^{\lambda_{1}}}\right)}^{\lambda }}{e^{e^{-\lambda_{2}/z^{\lambda_{1}}}}},\;z>0, \end{array}$$

with the corresponding PDF *k*(*z*; *λ*, Φ) given by

(11) $$\begin{array}{c} k\left(z;\lambda ,\Phi \right)=\frac{\lambda_{1}\lambda_{2}}{z^{\lambda_{1}+1}}e^{-\lambda_{2}/z^{\lambda_{1}}}{\left[1-e^{-\lambda_{2}/z^{\lambda_{1}}}\right]}^{\lambda -1}\frac{\left[\left(1+\lambda \right)-e^{-\lambda_{2}/z^{\lambda_{1}}}\right]}{e^{e^{-\lambda_{2}/z^{\lambda_{1}}}}},\;z>0. \end{array}$$

**Figure 3:**
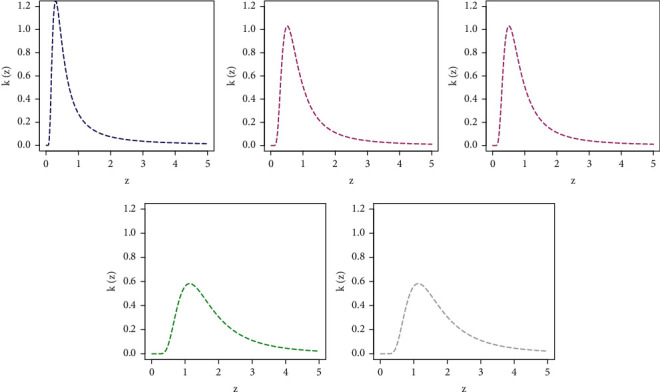
Different plots of *k*(*z*; *λ*, Φ).

To see the behavior of the NGI-Weibull model, different plots of *k*(*z*; *λ*, Φ) are obtained in Figure 3. The five different plots of *k*(*z*; *λ*, Φ) in Figure 3 are sketched for (a) *λ*_1_=1.2, *λ*_2_=0.5, *λ*=0.4 (blue line), (b) *λ*_1_=1.2, *λ*_2_=1, *λ*=1.2 (magenta line), (c) *λ*_1_=1.2, *λ*_2_=1.8, *λ*=1.6 (grey line), (d) *λ*_1_=1.2, *λ*_2_=2.8, *λ*=2.2 (green line), and (e) *λ*_1_=1.2, *λ*_2_=4.2, *λ*=2.8 (red line). From Figure 3, it is obvious that as the values of *λ* and *λ*_2_ increase, the NGI-Weibull captures the HT characteristics.

### 3.3. The HT Characteristics

Here, we use a mathematical approach to show that the NGI-Weibull model possesses the HT characteristics.

#### 3.3.1. Regular Variational Property

According to Seneta [31], in terms of SF [1 − *T*(*z*; Φ)], we have the following.

**Theorem 1 .**

If [1 − *T*(*z*; Φ)] is the SF of the RVD, then [1 − *K*(*z*; *λ*, Φ)] is a RVD.

**Proof**

Assume lim_*z*⟶*∞*_([1 − *T*(*mz*; Φ)]/[1 − *T*(*z*; Φ)])=*u*(*m*) is finite but nonzero for every *m* > 0. Incorporating equation (8), we get

(12) $$\begin{array}{c} \underset{z⟶\infty }{\text{lim}}\;\frac{1-K\left(mz;\lambda ,\Phi \right)}{1-K\left(z;\lambda ,\Phi \right)}=\;\underset{z⟶\infty }{\text{lim}}\frac{{\left[1-T\left(mz;\Phi \right)\right]}^{\lambda }}{{\left[1-T\left(z;\Phi \right)\right]}^{\lambda }}\cdot e^{T\left(z;\Phi \right)}/e^{T\left(mz;\Phi \right)},\\ \underset{z⟶\infty }{\text{lim}}\frac{1-K\left(mz;\lambda ,\Phi \right)}{1-K\left(z;\lambda ,\Phi \right)}=\;\underset{z⟶\infty }{\text{lim}}{\left(\frac{\left[1-T\left(mz;\Phi \right)\right]}{\left[1-T\left(z;\Phi \right)\right]}\right)}^{\lambda }\cdot e^{T\left(z;\Phi \right)}/e^{T\left(mz;\Phi \right)}. \end{array}$$

Using equation (6) in equation (12), we get

(13) $$\begin{array}{c} \underset{z⟶\infty }{\text{lim}}\frac{1-K\left(mz;\lambda ,\Phi \right)}{1-K\left(z;\lambda ,\Phi \right)}=\;\underset{z⟶\infty }{\text{lim}}{\left(\frac{\left[1-T\left(mz;\Phi \right)\right]}{\left[1-T\left(z;\Phi \right)\right]}\right)}^{\lambda }\cdot e^{e^{-\lambda_{2}/z^{\lambda_{1}}}}/e^{e^{-\lambda_{2}/{\left(mz\right)}^{\lambda_{1}}}},\\ \underset{z⟶\infty }{\text{lim}}\frac{1-K\left(mz;\lambda ,\Phi \right)}{1-K\left(z;\lambda ,\Phi \right)}=\;\underset{z⟶\infty }{\text{lim}}{\left(\frac{\left[1-T\left(mz;\Phi \right)\right]}{\left[1-T\left(z;\Phi \right)\right]}\right)}^{\lambda }\cdot e^{e^{-\lambda_{2}/\infty^{\lambda_{1}}}}/e^{e^{-\lambda_{2}/{\left(m.\infty \right)}^{\lambda_{1}}}},\\ \underset{z⟶\infty }{\text{lim}}\frac{1-K\left(mz;\lambda ,\Phi \right)}{1-K\left(z;\lambda ,\Phi \right)}=\;\underset{z⟶\infty }{\text{lim}}{\left(\frac{\left[1-T\left(mz;\Phi \right)\right]}{\left[1-T\left(z;\Phi \right)\right]}\right)}^{\lambda }\cdot e^{e^{-\lambda_{2}/\infty }}/e^{e^{-\lambda_{2}/\infty }},\\ \underset{z⟶\infty }{\text{lim}}\frac{1-K\left(mz;\lambda ,\Phi \right)}{1-K\left(z;\lambda ,\Phi \right)}=\;\underset{z⟶\infty }{\text{lim}}{\left(\frac{\left[1-T\left(mz;\Phi \right)\right]}{\left[1-T\left(z;\Phi \right)\right]}\right)}^{\lambda }\cdot e^{e^{0}}/e^{e^{0}},\\ \underset{z⟶\infty }{\text{lim}}\frac{1-K\left(mz;\lambda ,\Phi \right)}{1-K\left(z;\lambda ,\Phi \right)}=\;\underset{z⟶\infty }{\text{lim}}{\left(\frac{\left[1-T\left(mz;\Phi \right)\right]}{\left[1-T\left(z;\Phi \right)\right]}\right)}^{\lambda },\\ \underset{z⟶\infty }{\text{lim}}\frac{1-K\left(mz;\lambda ,\Phi \right)}{1-K\left(z;\lambda ,\Phi \right)}={\left(u\left(m\right)\right)}^{/}, \end{array}$$

where (*u*(*m*))^/^=lim_*z*⟶*∞*_([1 − *T*(*mz*; Φ)]/[1 − *T*(*z*; Φ)])^*λ*^.

Then, equation (13) is nonzero for every *m* > 0. Therefore, [1 − *K*(*z*; *λ*, Φ)] is the SF of the RVD.

#### 3.3.2. A Supportive Example of RVP

Consider *Z* has a power-law behavior, and then, according to the definition of the HT property, we have

(14) $$\begin{array}{c} 1-T\left(z;\Phi \right)=\mathbb{P}\left(Z>z\right)∼z^{-\alpha },\\ \text{or}\\ {\left[1-T\left(z;\Phi \right)\right]}^{\lambda }=\mathbb{P}\left(Z>z\right)∼z^{-\alpha }. \end{array}$$

Using the results of Karamata's characterization theorem [31], we can write 1 − *K*(*z*; *λ*, Φ) as follows:

(15) $$\begin{array}{c} 1-K\left(z;\lambda ,\Phi \right)=z^{-\alpha }L\left(z\right), \end{array}$$

where *L*(*z*) represents the slowly varying function (SVF). From (8), we have

(16) $$\begin{array}{c} 1-K\left(z;\lambda ,\Phi \right)=\frac{{\left[1-T\left(z;\Phi \right]\right)}^{\lambda }}{e^{T\left(z;\Phi \right)}},\\ 1-K\left(z;\lambda ,\Phi \right)=\frac{z^{-\alpha }}{e^{T\left(z;\Phi \right)}},\\ 1-K\left(z;\lambda ,\Phi \right)=\frac{z^{-\alpha }}{e^{T\left(z;\Phi \right)}},\\ 1-K\left(z;\lambda ,\Phi \right)=z^{-\alpha }L\left(z\right), \end{array}$$

where *L*(*z*)=(1/*e*^*T*(*z*; Φ)^). So, if we are able to show that *L*(*z*) is a SVF, then the variational result obtained in (16) is true. To prove that *L*(*z*) is SVF, we have shown that

(17) $$\begin{array}{c} \underset{z⟶\infty }{\text{lim}}\frac{L\left(az\right)}{L\left(z\right)}=1. \end{array}$$

So,

(18) $$\begin{array}{c} \frac{L\left(az\right)}{L\left(z\right)}=\frac{e^{T\left(z;\Phi \right)}}{e^{T\left(az;\Phi \right)}},\\ \frac{L\left(az\right)}{L\left(z\right)}=\frac{e^{e^{-\lambda_{2}/z^{\lambda_{1}}}}}{e^{e^{-\lambda_{2}/a^{\lambda_{1}}z^{\lambda_{1}}}}}. \end{array}$$

Applying the limit, we get

(19) $$\begin{array}{c} \underset{z⟶\infty }{\text{lim}}\frac{L\left(az\right)}{L\left(z\right)}=\frac{e^{e^{-\lambda_{2}/\infty^{\lambda_{1}}}}}{e^{e^{-\lambda_{2}/a^{\lambda_{1}}\infty^{\lambda_{1}}}}},\\ \underset{z⟶\infty }{\text{lim}}\frac{L\left(az\right)}{L\left(z\right)}=\frac{e^{e^{0}}}{e^{e^{0}}},\\ \underset{z⟶\infty }{\text{lim}}=\frac{L\left(az\right)}{L\left(z\right)}=1. \end{array}$$

### 3.4. Mathematical Properties

Here, we derive some mathematical properties of the NGI-Weibull distribution.

#### 3.4.1. Asymptotics

Note that 1 − (1 − *x*)^*a*^ ~ *ax* as *x*⟶0, by taking *x*=*e*^−*λ*_2_ *z*^−*λ*_1_^^, the asymptotics of (10) and (11) as *z*⟶0 are given by

(20) $$\begin{array}{c} K\left(z\right)∼\lambda e^{-\lambda_{2}z^{-\lambda_{1}}},\\ k\left(z\right)∼\lambda \lambda_{1}\lambda_{2}z^{-\lambda_{1}-1}e^{-\lambda_{2}z^{-\lambda_{1}}} \end{array}$$

respectively.

Note that 1 − *e*^−*x*^ ~ *x* as *x* ~ 0, by taking *x*=*e*^−*λ*_2_ *z*^−*λ*_1_^^, the asymptotics of (10) and (11) as *z*⟶*∞* are given by

(21) $$\begin{array}{c} 1-K\left(z\right)∼\lambda_{2}^{\lambda }e^{z^{-\lambda \lambda_{1}}},\\ k\left(z\right)∼\lambda \lambda_{1}\lambda_{2}^{\lambda }e^{z^{-\lambda \lambda_{1}-1}} \end{array}$$

respectively.

#### 3.4.2. Moments and Incomplete Moments

Let *Z* follows the NGI-Weibull with parameters (*λ*, *λ*_1_, *λ*_2_), and then, the *n*^th^ moment of *Z* is given by

(22) $$\begin{array}{c} E\left(Z^{n}\right)=\int_{0}^{\infty }z^{n}k\left(z\right)\text{d}z. \end{array}$$

After using generalized binomial expansion and the Taylor expansion, we can obtain

(23) $$\begin{array}{c} E\left(Z^{n}\right)=\sum_{i,j=0}^{\infty }\frac{{\left(-1\right)}^{i+j}\left(\begin{array}{c} \alpha -1\\ i \end{array}\right)}{j!}\left(\lambda +1\right)\lambda_{1}\lambda_{2}\int_{0}^{\infty }z^{n-\lambda_{1}-1}e^{-\lambda_{2}\left(i+j+1\right)z^{-\lambda_{1}}}\text{ d}z\\ +\sum_{i,j=0}^{\infty }\frac{{\left(-1\right)}^{i+j}\left(\begin{array}{c} \alpha -1\\ i \end{array}\right)}{j!}\left(\lambda -1\right)\lambda_{1}\lambda_{2}\int_{0}^{\infty }z^{n-\lambda_{1}-1}e^{-\lambda_{2}\left(i+j+2\right)z^{-\lambda_{1}}}\text{ d}z,\\ E\left(Z^{n}\right)=\sum_{i,j=0}^{\infty }\frac{{\left(-1\right)}^{i+j}\left(\begin{array}{c} \alpha -1\\ i \end{array}\right)}{j!}\left(\lambda +1\right)\lambda_{1}\lambda_{2}\frac{\Gamma \left(\left(1-n\right)/\lambda_{1}\right)}{{\left(\lambda_{2}\left(i+j+1\right)\right)}^{\left(1-n\right)/\lambda_{1}}}\\ +\sum_{i,j=0}^{\infty }\frac{{\left(-1\right)}^{i+j}\left(\begin{array}{c} \alpha -1\\ i \end{array}\right)}{j!}\left(\lambda -1\right)\lambda_{1}\lambda_{2}\frac{\Gamma \left(\left(1-n\right)/\lambda_{1}\right)}{{\left(\lambda_{2}\left(i+j+2\right)\right)}^{\left(1-n\right)/\lambda_{1}}}. \end{array}$$

For incomplete moments, we have

(24) $$\begin{array}{c} E\left(Z^{n}|Z\leq z\right)=\frac{1}{K\left(z\right)}\int_{0}^{z}t^{n}k\left(t\right)\text{d}t. \end{array}$$

After using generalized binomial expansion and the Taylor expansion, we obtain

(25) $$\begin{array}{c} E\left(Z^{n}|Z\leq z\right)=\frac{1}{K\left(z\right)}\sum_{i,j=0}^{\infty }\frac{{\left(-1\right)}^{i+j}\left(\begin{array}{c} \alpha -1\\ i \end{array}\right)}{j!}\left(\lambda +1\right)\lambda_{1}\lambda_{2}\int_{0}^{z}t^{n-\lambda_{1}-1}e^{-\lambda_{2}\left(i+j+1\right)t^{-\lambda_{1}}}\text{ d}t\\ +\frac{1}{K\left(z\right)}\sum_{i,j=0}^{\infty }\frac{{\left(-1\right)}^{i+j}\left(\begin{array}{c} \alpha -1\\ i \end{array}\right)}{j!}\left(\lambda -1\right)\lambda_{1}\lambda_{2}\int_{0}^{z}t^{n-\lambda_{1}-1}e^{-\lambda_{2}\left(i+j+2\right)t^{-\lambda_{1}}}\text{ d}t. \end{array}$$

Using *t*^−*λ*_1_^=*u* transformation, we obtain

(26) $$\begin{array}{c} E\left(Z^{n}|Z\leq z\right)=\frac{1}{K\left(z\right)}=\sum_{i,j=0}^{\infty }\frac{{\left(-1\right)}^{i+j}\left(\begin{array}{c} \alpha -1\\ i \end{array}\right)}{j!}\left(\lambda +1\right)\lambda_{1}\lambda_{2}\frac{\int_{\lambda_{2}\left(i+j+1\right)z^{-\lambda_{1}}}^{\infty }u^{\left(\left(1-n\right)/\lambda_{1}\right)-1}e^{-u}\text{d}u}{{\left(\lambda_{2}\left(i+j+1\right)\right)}^{\left(1-n\right)/\lambda_{1}}}\\ +\frac{1}{K\left(z\right)}\sum_{i,j=0}^{\infty }\frac{{\left(-1\right)}^{i+j}\left(\begin{array}{c} \alpha -1\\ i \end{array}\right)}{j!}\left(\lambda -1\right)\lambda_{1}\lambda_{2}\frac{\int_{\lambda_{2}\left(i+j+2\right)z^{-\lambda_{1}}}^{\infty }u^{\left(\left(1-n\right)/\lambda_{1}\right)-1}e^{-u}\text{ d}u}{{\left(\lambda_{2}\left(i+j+2\right)\right)}^{\left(1-n\right)/\lambda_{1}}},\\ E\left(Z^{n}|Z\leq z\right)=\frac{1}{K\left(z\right)}\sum_{i,j=0}^{\infty }\frac{{\left(-1\right)}^{i+j}\left(\begin{array}{c} \alpha -1\\ i \end{array}\right)}{j!}\left(\lambda +1\right)\lambda_{1}\lambda_{2}\frac{\Gamma \left(\left(1-n\right)/\lambda_{1},\lambda_{2}\left(i+j+1\right)z^{-\lambda_{1}}\right)}{{\left(\lambda_{2}\left(i+j+1\right)\right)}^{\left(1-n\right)/\lambda_{1}}}\\ +\frac{1}{K\left(z\right)}\sum_{i,j=0}^{\infty }\frac{{\left(-1\right)}^{i+j}\left(\begin{array}{c} \alpha -1\\ i \end{array}\right)}{j!}\left(\lambda -1\right)\lambda_{1}\lambda_{2}\frac{\Gamma \left(\left(1-n\right)/\lambda_{1},\lambda_{2}\left(i+j+2\right)z^{-\lambda_{1}}\right)}{{\left(\lambda_{2}\left(i+j+2\right)\right)}^{\left(1-n\right)/\lambda_{1}}}, \end{array}$$

where Γ(*s*, *z*)=∫_*z*_^*∞*^*u*^*s*−1^*e*^−*u*^ d*u* is the upper incomplete gamma function.

#### 3.4.3. Order Statistics

Let *Z*_1_, *Z*_2_,…, *Z*_*n*_ be random variables with size *n* from (10); then, the DF of *i*^th^ order statistics (OS) is given by

(27) $$\begin{array}{c} K_{i:n}\left(z\right)=\sum_{l=i}^{n}\left(\begin{array}{c} n\\ l \end{array}\right)K{\left(z\right)}^{l}{\left(1-K\left(z\right)\right)}^{n-l},\\ K_{i:n}\left(z\right)=\sum_{l=i}^{n}\sum_{j=0}^{l}{\left(-1\right)}^{j}\left(\begin{array}{c} l\\ j \end{array}\right)\left(\begin{array}{c} n\\ l \end{array}\right){\left[1-K\left(z\right)\right]}^{n-l+j},\\ \text{or}\\ K_{i:n}\left(z\right)=\sum_{l=i}^{n}\sum_{j=0}^{l}{\left(-1\right)}^{j}\left(\begin{array}{c} l\\ j \end{array}\right)\left(\begin{array}{c} n\\ l \end{array}\right)e^{-\left(n-l+j\right)e^{-\lambda_{2}z^{-\lambda_{1}}}}{\left[1-e^{-\lambda_{2}z^{-\lambda_{1}}}\right]}^{\lambda \left(n-l+j\right)}. \end{array}$$

By differentiating equation (27), we get the PDF of the *i*^th^ OS as given by

(28) $$\begin{array}{c} k_{i:n}\left(z\right)=\frac{d}{dz}K_{i:n}\left(z\right). \end{array}$$

### 3.5. Estimation

Here, we adopt different estimation methods to derive the estimators $\left(\hat{\lambda },\hat{\lambda_{1}},\hat{\lambda_{2}}\right)$ of the parameters (*λ*, *λ*_1_, *λ*_2_) of the NGI-Weibull distribution.

#### 3.5.1. Maximum-Likelihood Estimation

Consider a random sample (RS) as *Z*_1_, *Z*_2_,…, *Z*_*p*_ taken from *k*(*z*; *λ*, Φ). Corresponding to *k*(*z*; *λ*, Φ), the log-likelihood (LL) function Δ(*λ*, Φ) is given by

(29) $$\begin{array}{c} \Delta \left(\lambda ,\Phi \right)=p\;\log\;\lambda_{1}+p\;\log\;\lambda_{2}-\left(\lambda_{1}+1\right)\sum_{v=1}^{p}\log\;z_{v}-\lambda_{2}\sum z_{v}^{-\lambda_{1}}-\sum_{v=1}^{p}e^{-\lambda_{2}z_{v}^{-\lambda_{1}}}\\ +\left(\lambda_{1}-1\right)\sum_{v=1}^{p}\log\left[1-e^{-\lambda_{2}z_{v}^{-\lambda_{1}}}\right]+\sum_{v=1}^{p}\log\left[\left(1+\lambda \right)-e^{-\lambda_{2}z_{v}^{-\lambda_{1}}}\right]. \end{array}$$

With respect to *λ*, *λ*_2_, and *λ*_2_, the partial derivatives of Δ(*λ*, Φ) are given by

(30) $$\begin{array}{c} \frac{\partial }{\partial \lambda }\Delta \left(\lambda ,\Phi \right)=\sum_{v=1}^{p}\frac{1}{\left[\left(1+\lambda \right)-e^{-\lambda_{2}z_{v}^{-\lambda_{1}}}\right]},\\ \frac{\partial }{\partial \lambda_{1}}\Delta \left(\lambda ,\Phi \right)=\frac{p}{\lambda_{1}}-\sum_{v=1}^{p}\log z_{v}+\lambda_{2}\sum \log\left(z_{v}\right)z_{v}^{-\lambda_{1}}-\sum_{v=1}^{p}\frac{\log\left(\lambda_{2}z_{v}^{-\lambda_{1}}\right)\lambda_{2}z_{v}^{-\lambda_{1}}e^{-\lambda_{2}z_{v}^{-\lambda_{1}}}}{\left[\left(1+\lambda \right)-e^{-\lambda_{2}z_{v}^{-\lambda_{1}}}\right]}\\ +\sum_{v=1}^{p}\left(\log\left[1-e^{-\lambda_{2}z_{v}^{-\lambda_{1}}}\right]-\frac{\log\left(\lambda_{2}z_{v}^{-\lambda_{1}}\right)\left(\lambda_{1}-1\right)\lambda_{2}z_{v}^{-\lambda_{1}}e^{-\lambda_{2}z_{v}^{-\lambda_{1}}}}{\left[1-e^{-\lambda_{2}z_{v}^{-\lambda_{1}}}\right]}\right)\\ -\sum_{v=1}^{p}\log\left(\lambda_{2}z_{v}^{-\lambda_{1}}\right)\left(\lambda_{2}z_{v}^{-\lambda_{1}}\right)e^{-\lambda_{2}z_{v}^{-\lambda_{1}}},\\ \frac{\partial }{\partial \lambda_{2}}\Delta \left(\lambda ,\Phi \right)=\frac{p}{\lambda_{2}}-\sum z_{v}^{-\lambda_{1}}+\sum_{v=1}^{p}z_{v}^{-\lambda_{1}}e^{-\lambda_{2}z_{v}^{-\lambda_{1}}}+\left(\lambda_{1}-1\right)\sum_{v=1}^{p}\frac{z_{v}^{-\lambda_{1}}e^{-\lambda_{2}z_{v}^{-\lambda_{1}}}}{\left[1-e^{-\lambda_{2}z_{v}^{-\lambda_{1}}}\right]}\\ +\sum_{v=1}^{p}\frac{z_{v}^{-\lambda_{1}}e^{-\lambda_{2}z_{v}^{-\lambda_{1}}}}{\left[\left(1+\lambda \right)-e^{-\lambda_{2}z_{v}^{-\lambda_{1}}}\right]}, \end{array}$$

respectively.

On solving (∂/∂*λ*)Δ(*λ*, Φ)=0, (∂/∂*λ*_1_)Δ(*λ*, Φ)=0, and (∂/∂*λ*_2_)Δ(*λ*, Φ)=0, we get the MLEs $\left(\hat{\lambda },\hat{\lambda_{1}},\hat{\lambda_{2}}\right)$ of the parameters (*λ*, *λ*_1_, *λ*_2_).

#### 3.5.2. The Least-Squares and Weighted Least-Squares Estimation Methods

Here, we derive the least-squares estimators (LSEs) and weighted least-squares estimators (WLSEs) of the NGI-Weibull. Let {*s*_*i*_; *i*=1,2,…, *n*} be a RS and {*s*_*i*:*n*_; *i*=1,2,…, *n*} be the associated order statistics, and *K*(.) is the DF of NGI-Weibull. Then, the LSEs of the NGI-Weibull are obtained by solving equations:

(31) $$\begin{array}{c} \frac{\partial S_{\text{LSE}}\left(\lambda ,\lambda_{1},\lambda_{2}\right)}{\partial \lambda }=0,\\ \frac{\partial S_{\text{LSE}}\left(\lambda ,\lambda_{1},\lambda_{2}\right)}{\partial \lambda_{1}}=0,\\ \frac{\partial S_{\text{LSE}}\left(\lambda ,\lambda_{1},\lambda_{2}\right)}{\partial \lambda_{2}}=0, \end{array}$$

where

(32) $$\begin{array}{c} S_{\text{LSE}}\left(\lambda ,\lambda_{1},\lambda_{2}\right)=\sum_{i=1}^{n}{\left(K_{\text{NGI}-\text{Weibul}}\left(s_{i:n};\lambda ,\lambda_{1},\lambda_{2}\right)-\frac{i}{n+1}\right)}^{2}. \end{array}$$

The WLSEs of the NGI-Weibull distribution are obtained by solving equations:

(33) $$\begin{array}{c} \frac{\partial S_{\text{WLSE}}\left(\lambda ,\lambda_{1},\lambda_{2}\right)}{\partial \lambda }=0,\\ \frac{\partial S_{\text{WLSE}}\left(\lambda ,\lambda_{1},\lambda_{2}\right)}{\partial \lambda_{1}}=0,\\ \frac{\partial S_{\text{WLSE}}\left(\lambda ,\lambda_{1},\lambda_{2}\right)}{\partial \lambda_{2}}=0, \end{array}$$

where

(34) $$\begin{array}{c} S_{\text{WLSE}}\left(\lambda ,\lambda_{1},\lambda_{2}\right)=\sum_{i=1}^{n}\frac{{\left(n+1\right)}^{2}\left(n+2\right)}{i\left(n-i+1\right)}{\left(K_{\text{NGI}-\text{Weibul}}\left(s_{i:n};\lambda ,\lambda_{1},\lambda_{2}\right)-\frac{i}{n+1}\right)}^{2}. \end{array}$$

#### 3.5.3. Cramér–von Mises Estimator

Here, we obtain the Cramér–von Mises estimators (CMEs) of the NGI-Weibull distribution. The CMEs are obtained by minimizing the following functions:

(35) $$\begin{array}{c} \frac{\partial S_{\text{CME}}\left(\lambda ,\lambda_{1},\lambda_{2}\right)}{\partial \lambda }=0,\\ \frac{\partial S_{\text{CME}}\left(\lambda ,\lambda_{1},\lambda_{2}\right)}{\partial \lambda_{1}}=0,\\ \frac{\partial S_{\text{CME}}\left(\lambda ,\lambda_{1},\lambda_{2}\right)}{\partial \lambda_{2}}=0, \end{array}$$

where

(36) $$\begin{array}{c} S_{\text{CME}}\left(\lambda ,\lambda_{1},\lambda_{2}\right)=\frac{1}{12n}+\sum_{i=1}^{n}{\left(K_{\text{NGI}-\text{Weibul}}\left(s_{i:n};\lambda ,\lambda_{1},\lambda_{2}\right)-\frac{2i-1}{2n}\right)}^{2}. \end{array}$$

### 3.6. Application to Sales Data

This subsection deals with the implementation of the NGI-Weibull distribution to Instagram advertising data given by 11.451, 6.357, 5.186, 7.363, 6.765, 3.683, 7.343, 5.190, 3.009, 6.398, 5.178, 7.638, 6.240, 5.643, 8.846, 11.673, 5.243, 10.130, 6.725, 8.010, 7.610, 4.479, 17.753, 9.932, 5.634, 6.151, 7.989, 6.826, 10.195, 6.006, 11.265, 7.004, 5.477, 8.616, 5.029, 6.600, 13.140, 6.733, 5.594, 11.294, 7.121, 9.427, 10.728, 7.218, 4.940, 6.648, 4.479, 11.811, 7.945, 4.079, 6.814, 5.388, 9.145, 11.609, 11.661, 12.514, 3.671, 7.452, 9.028, 9.157, 4.580, 13.188, 9.028, 7.977, 10.070, 5.796, 5.614, 6.196, 11.011, 8.038, 9.472, 6.547, 3.546, 4.839, 9.068, 5.566, 2.415, 5.388, 3.473, 4.625, 6.770, 7.367, 4.645, 5.647, 9.383, 7.892, 7.137, 8.575, 7.630, 8.922, 6.018, 3.732, 9.912, 11.015, 5.691, 8.620, 6.321, 7.989, 12.614, 8.967, 6.248, 12.017, 7.767, 7.569, 10.441, 9.730, 3.821, 5.117, 2.968, and 10.090. Corresponding to these data, the basic measures (BMs) are as follows: minimum = 2.415, 1st quartile = 5.599, 3rd quartile = 9.154, range = 15.338, median = 7.178, mean = 7.539, variance = 7.311, standard deviation = 2.704, skewness = 0.669, kurtosis = 3.662, and maximum = 17.753.

The NGI-Weibull is applied to Instagram advertising data to establish its flexibility and best fitting capability. For this practical demonstration, the results of the NGI-Weibull model are compared with the parent model (IW distribution) and exponentiated Lomax (Exp-Lomax) distributions.

Singh et al. [32] showed that the IW model fits the financial data sets closely than the other well-known competitors. The Exp-Lomax is a flexible modification of the Lomax model, which was basically introduced for dealing with data in the finance sector. The survival functions (SFs) of the competitive distributions are given by

(i) The IW model
  (37) $$\begin{array}{c} S\left(z;\Phi \right)=1-e^{-\lambda_{2}/z^{\lambda_{1}}},\;z,\lambda_{1},\lambda_{2}>0. \end{array}$$
(ii) The Exp-Lomax model
  (38) $$\begin{array}{c} S\left(z;\beta ,\Phi \right)=1-{\left(1-{\left[1+\lambda_{2}z\right]}^{-\lambda_{1}}\right)}^{\beta },\;z,\lambda_{1},\lambda_{2},\beta >0. \end{array}$$

**Figure 4:**
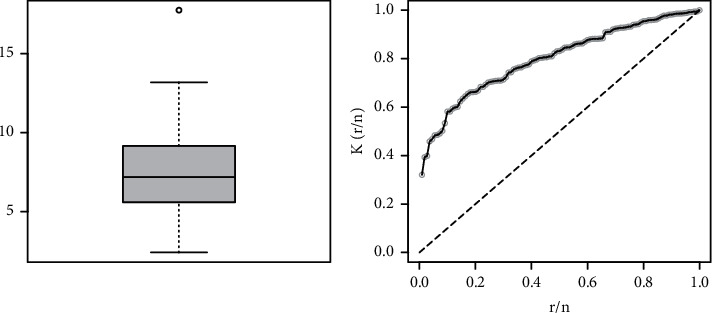
The BP and TTT plot of the Instagram sales data.

The graphs of boxplot (BP) and total time test (TTT) for the Instagram sales data are provided in Figure 4.

To show the usefulness of the NGI-Weibull model, certain statistical tools (STs) are considered. These STs consist of four information criteria (IC) and three goodness-of-fit measures (GFMs) along with the *p* value. The values of the IC are calculated as follows:

(i) The Akaike IC (AIC)
  (39) $$\begin{array}{c} \text{AIC}=2m-2\Delta \left(\Phi \right), \end{array}$$
(ii) The Bayesian IC (BIC)
  (40) $$\begin{array}{c} \text{BIC}=m\;\log\left(p\right)-2\Delta \left(\Phi \right), \end{array}$$
(iii) The corrected AIC (CAIC)
  (41) $$\begin{array}{c} \text{CAIC}=\frac{2mp}{p-m-1}-2\Delta \left(\Phi \right), \end{array}$$
(iv) The Hannan–Quinn IC (HQIC)
  (42) $$\begin{array}{c} \text{HQIC}=2m\;\log\left(\log\left(p\right)\right)-2\Delta \left(\Phi \right), \end{array}$$
where the terms *p*, *n*, and Δ(Φ) represent the number of parameters, sample size, and LL function, respectively.

The values of the GFMs are calculated as follows:

(i) The Anderson–Darling (AD) test statistic
  (43) $$\begin{array}{c} \text{AD}=-p-\frac{1}{p}\sum_{v=1}^{p}\left(2v-1\right)\left[\log\;K\left(z_{v}\right)+\log\left\{1-K\left(z_{p-v+1}\right)\right\}\right]. \end{array}$$
(ii) The Cramér–von Mises (CM) test
  (44) $$\begin{array}{c} \text{CM}=\frac{1}{12p}+\sum_{v=1}^{p}{\left[\frac{2v-1}{2p}-K\left(z_{v}\right)\right]}^{2}. \end{array}$$
(iii) The Kolmogorov–Smirnov (KS) test
  (45) $$\begin{array}{c} \text{KS}={\text{sup}}_{z}\left[K_{p}\left(z\right)-K\left(z\right)\right]. \end{array}$$

Corresponding to the Instagram sales data, the numerical estimates (NEs) of the model parameters are obtained via implementing the *R-script* with the method *SANN*; see Appendix. The NEs of the fitted models are provided in Table 3, whereas the values of IC measures and GFMs of the fitted models are, respectively, given in Tables 4 and 5.

For the underline data, a model with a larger *p* value and smaller values of IC and GFMs is considered a better model. From the presented results in Tables 4 and 5, it is obvious that the NGI-Weibull model is the best, as it has the smallest values of the IC and GFMs and a larger *p* value. This fact reveals the applicability and importance of the NGI-Weibull distribution to deal with Instagram sales data and other data sets in the business management and finance sectors.

**Figure 5:**
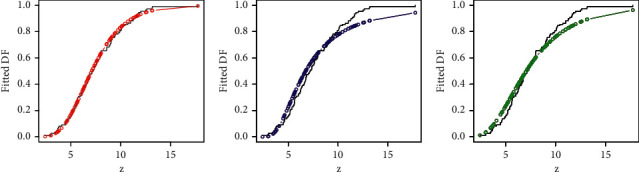
Corresponding to Instagram sales data, the fitted DFs of the models.

**Figure 6:**
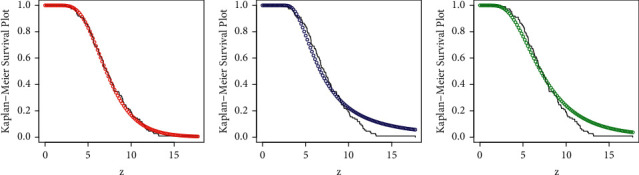
Corresponding to Instagram sales data, the fitted SFs of the models.

**Figure 7:**
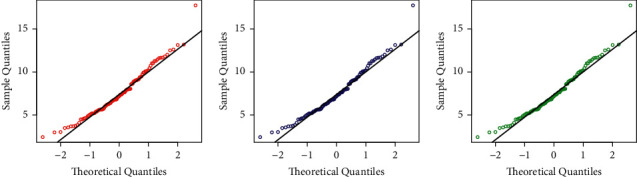
Corresponding to Instagram sales data, the QQ plots of the models.

**Figure 8:**
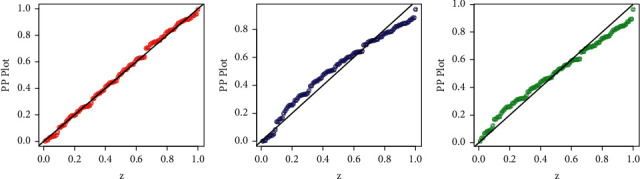
Corresponding to Instagram sales data, the PP plots of the models.

Besides the numerical illustration, a visual display of the performances of the competing models is presented in Figures 5–8. For the visual comparison, we plotted the graphs of the fitted DFs (Figure 5), SFs (Figure 6), QQ (Figure 7), and PP (Figure 8) of the fitted models. It is important to note that the plots in Figures 5–8 are obtained for NGI-Weibull (red line), IW (blue line), and Exp-Lomax (green line).

## 4. Concluding Remarks

This work explored the impact of online marketing on sales. Among the available online marketing media, a well-known online medium called Instagram is considered. The data sets related to Instagram advertising and sales were studied and analyzed scientifically. To carry out the analysis, we implemented the linear regression approach along with the *F*-test and *t*-test. Based on these tests, it showed that there is a positive impact of Instagram advertising on sales. According to the finding of this study, it showed that spending money on Instagram advertising can increase the sale. In addition to the *F*-test and *t*-test, we also performed the CT. Based on the results of the CT, it was observed that there is a positive correlation between Instagram advertising and sales.

Finally, a new statistical model named a NGI-Weibull was introduced and studied in detail. Certain mathematical properties along the HT characteristics of the NGI-Weibull distribution were obtained. The NGI-Weibull was applied to model the Instagram advertising sales data. The comparison of the NGI-Weibull model was made with the other models. Certain statistical tools (AIC, CM, BIC, AD, CAIC, KS, and HQIC) were considered for comparative purposes to see which model provides the best description of the Instagram advertising sales data. Using these statistical tools, it showed that the NGI-Weibull model is the best model for taking care of financial data sets.

## Figures and Tables

**Table 1:** Regression analysis using Instagram advertising and sales data.

Adv. media		Coefficients		Esti. values		Standard error		*t*-statistic		Pr (>*|t|*)	
YouTube		*λ*_0_		5.1030		0.44807		11.389		2*e* − 16	
		*λ*_1_		0.1935		0.03092		6.259		8.01*e* − 09	

**Table 2:** Regression analysis using Instagram advertising medium.

Adv. media		*R* ^2^		Adjusted *R*^2^		*F*-statistic		*p* value		Degree of freedom	
YouTube		0.2662		0.2594		39.17		8.011e-09		1 and 108	

**Table 3:** The values of the estimated parameters of the fitted models.

Models	*λ*	*λ*_1_	*λ*_2_	*β*
NGI-Weibull	16.910 6	0.9307	20.1934	—
IW	—	2.5589	91.4791	—
Exp-Lomax	—	7.9471	0.0578	10.1876

**Table 4:** The numerical values of the IC measures of the fitted distributions.

Models	AIC	CAIC	BIC	HQIC
NGI-Weibull	528.2917	528.5181	536.3931	531.5776
IW	553.9948	554.1070	559.3958	556.1855
Exp-Lomax	544.1750	544.4014	552.2765	547.4610

**Table 5:** The GFMs of the fitted models.

Models	CM	AD	KS	*p* value
NGI-Weibull	0.0310	0.2742	0.0465	0.9707
IW	0.3138	2.1091	0.1078	0.1544
Exp-Lomax	0.0500	0.4164	0.0977	0.2438

## Data Availability

The data are available from the corresponding author upon request.

## Appendix

The R-code is used for analysis under Section 3.

data=c(11.451, 6.357, 5.186, 7.363, 6.765, 3.683, 7.343, 5.190, 3.009,
6.398, 5.178, 7.638, 6.240, 5.643, 8.846, 11.673, 5.243, 10.130, 6.725,
8.010, 7.610, 4.479, 17.753, 9.932, 5.634, 6.151, 7.989, 6.826, 10.195,
6.006, 11.265, 7.004, 5.477, 8.616, 5.029, 6.600, 13.140, 6.733, 5.594,
11.294, 7.121, 9.427, 10.728, 7.218, 4.940, 6.648, 4.479, 11.811, 7.945,
4.079, 6.814, 5.388, 9.145, 11.609, 11.661, 12.514, 3.671, 7.452, 9.028,
9.157, 4.580, 13.188, 9.028, 7.977, 10.070, 5.796, 5.614, 6.196, 11.011,
8.038, 9.472, 6.547, 3.546, 4.839, 9.068, 5.566, 2.415, 5.388, 3.473,
4.625, 6.770, 7.367, 4.645, 5.647, 9.383, 7.892, 7.137, 8.575, 7.630,
8.922, 6.018, 3.732, 9.912, 11.015, 5.691, 8.620, 6.321, 7.989, 12.614,
8.967, 6.248, 12.017, 7.767, 7.569, 10.441, 9.730, 3.821, 5.117, 2.968, 10.090)
################################################################################
##### PDF of the NGI-Weibull distribution
################################################################################
pdf_pm <- function(par,x)
{
lam= par[1]
lam1= par[2]
lam2= par[3]
lam1*∗*lam2*∗*(x^(-lam1-1))*∗*exp(-lam2/x^lam1)*∗*((1-exp(-lam2/x^lam1))^(lam-1))
*∗*((1+lam)-exp(-lam2/x^lam1))/exp(exp(-lam2/x^lam1))
}
################################################################################
##### DF of the NGI-Weibull distribution
################################################################################
cdf_pm <- function(par,x)
{
lam= par[1]
lam1= par[2]
lam2= par[3]
1-(((1-exp(-lam2/x^lam1))^(lam))/(exp(exp(-lam2/x^lam1))))
}
set.seed(0)
goodness.fit(pdf=pdf_pm, cdf=cdf_pm,
starts = c(0.5,0.5,0.5), data = data,
method=”SANN,” domain=c(0,Inf),mle=NULL)
